# Case Report: Drug-Induced (Neuro) Sarcoidosis-Like Lesion Under IL4 Receptor Blockade With Dupilumab

**DOI:** 10.3389/fneur.2022.881144

**Published:** 2022-06-20

**Authors:** Stergios Tsitos, Lisa Catherina Niederauer, Paula Albert i Gracenea, Johanna Mueller, Andreas Straube, Louisa Von Baumgarten

**Affiliations:** ^1^Department of Neurology, University Hospital, Ludwig-Maximilians-Universität (LMU) Munich, Munich, Germany; ^2^Department of Internal Medicine, Hospital Interlaken, Unterseen, Switzerland; ^3^Department of Neurosurgery, University Hospital, Ludwig-Maximilians-Universität (LMU) Munich, Munich, Germany

**Keywords:** neurosarcoidosis, immune-related side effect, dupilumab, IL-4 receptor alpha, drug-induced sarcoid-like reaction

## Abstract

Dupilumab is a new monoclonal antibody inhibiting IL-4 and IL-13 signaling transduction through the blockage of the α-subunit of the IL-4 receptor. It is used to treat type 2 inflammatory disorders including atopic dermatitis, asthma, and chronic rhinosinusitis. Here we describe the case of a 79-year-old male presenting with visual hallucinations, disorientation, cognitive decline, and behavioral changes, evolving over 3 weeks. He had been under treatment with dupilumab for atopic dermatitis for the previous 4 months. Radiology and CSF analysis showed a granulomatous meningoencephalitis suspicious of sarcoidosis. Underlying infectious and antibody-mediated causes for meningoencephalitis were ruled out. Pausing Dupilumab and steroids (i.v. and oral) led to rapid clinical improvement. Inhibition of IL-4 and IL-13, key players in the differentiation and activation of Th2 cells, may shift the Th1/Th2- ratio toward an excessive Th1-mediated response, granuloma formation, and drug-induced (neuro)sarcoidosis reaction. Attention should be raised to this side effect.

## Background

Dupilumab is a human monoclonal IgG4-antibody, that blocks the α-subunit of the IL-4 receptor (IL-4R α). Through IL-4R α blockage, the IL-13 and IL-4 signaling pathways are modulated, leading to a decrease in Th2-biomarkers (1, 2). Dupilumab has shown clinical activity in the treatment of type 2 inflammatory disorders like atopic dermatitis (AD), asthma, and chronic rhinosinusitis with nasal polyps (CRSwNP). It received approval from the United States Food and Drug Administration and the European Commission for moderate-to-severe atopic dermatitis and subsequently for asthma in 2018 and 2019, respectively (1–3).

The most common adverse reactions, reported so far, were local reactions at the injection site, conjunctivitis (2), and headache. Less frequently, nasopharyngitis, nausea, arthralgia, gastritis, insomnia, and toothache occurred (1, 2). However, with increased use, new nuances of the side effect spectrum may emerge.

## Case Presentation

Here, we report the case of a 79-year-old male patient presenting with visual hallucinations, disorientation, difficulty finding words, cognitive decline, and behavioral changes including aggression, as well as burning sensations in both legs and arms, which had gradually evolved over 3 weeks before admission. Upon examination of temporal disorientation, reduced reflex levels of the lower extremities, symmetrical loss of sensation on both feet until below the knees, rigorously increased tone of the upper extremities, predominantly on the right side, and an uncertain gait with small steps, and the patient leaning forward was recorded. The remainder neurologic exam was unremarkable with no headache being reported.

He was under treatment with dupilumab injections every 2 weeks and 6 mg of prednisolone daily for his atopic dermatitis for the past 4 months. Apart from AD, he had undergone two surgeries for his lumbar spinal stenosis and a lumbar disc protrusion during the past year and was also suffering from cervical spinal stenosis, which explained his reduced reflex levels, sensory deficits, burning sensation, and gait impairment. Apart from the rigorously increased tone of his upper limbs, he showed no other signs of parkinsonism, so this was treated as an incidental finding upon examination. He was under treatment with tamsulosin for his prostate adenoma, and atorvastatin for hyperlipidemia and had recently been started on a low dose of pregabalin for his presumed neuropathic pain. His family history was clear for neurologic conditions.

**Figure 1:**
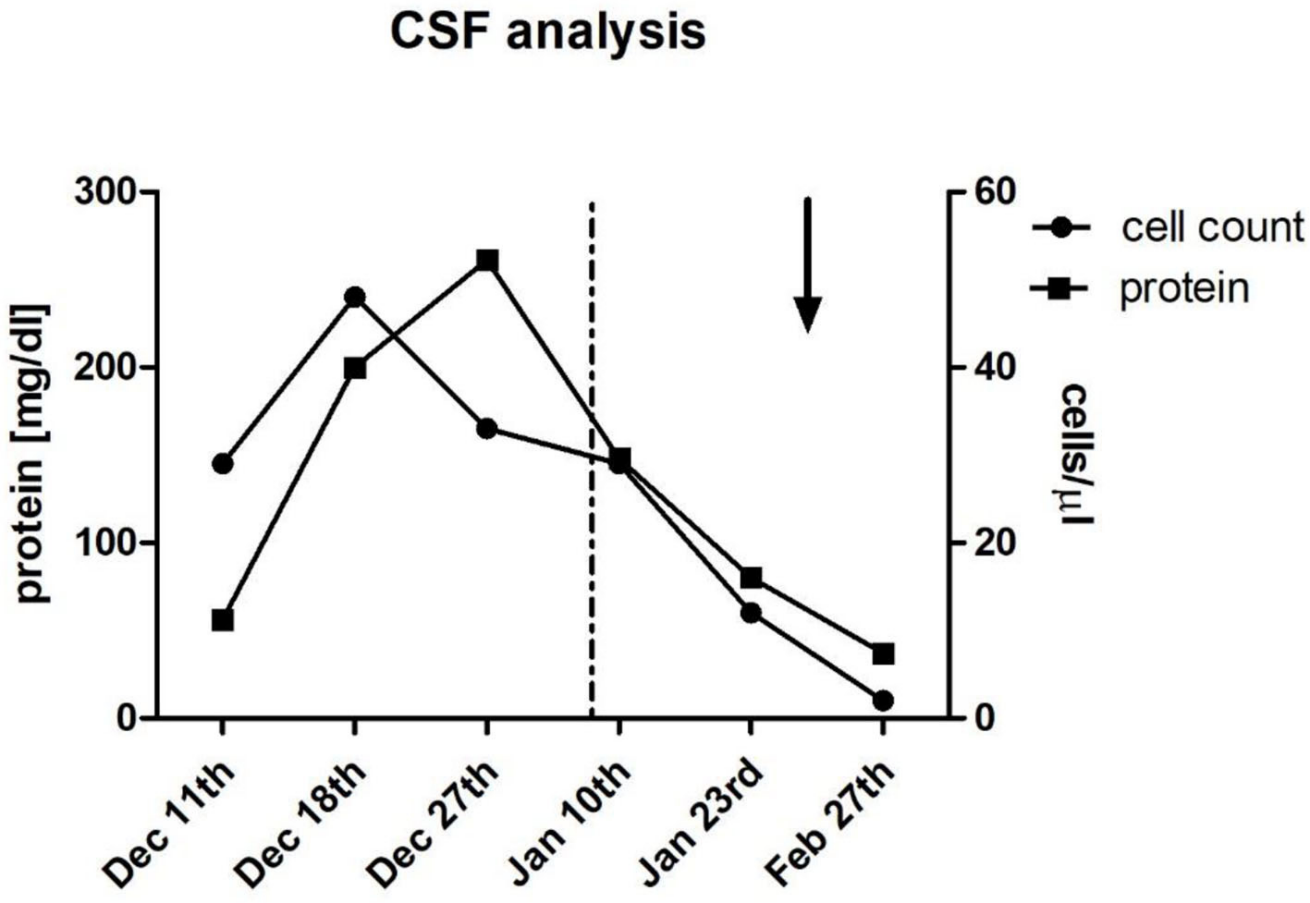
Cerebrospinal fluid (CSF) analysis. Multiple lumbar punctures during hospitalization and outpatient follow-up showed initial lymphocytic pleocytosis (up to 48 cells/μl) and elevated protein (up to 261 mg/dl) which improved after discontinuation of dupilumab (dotted line; Jan 8th), followed by administration of 1g Methylprednisolone IV for 3 days (arrow; Jan 27th), and subsequent oral steroid treatment for 4 weeks.

**Figure 2:**
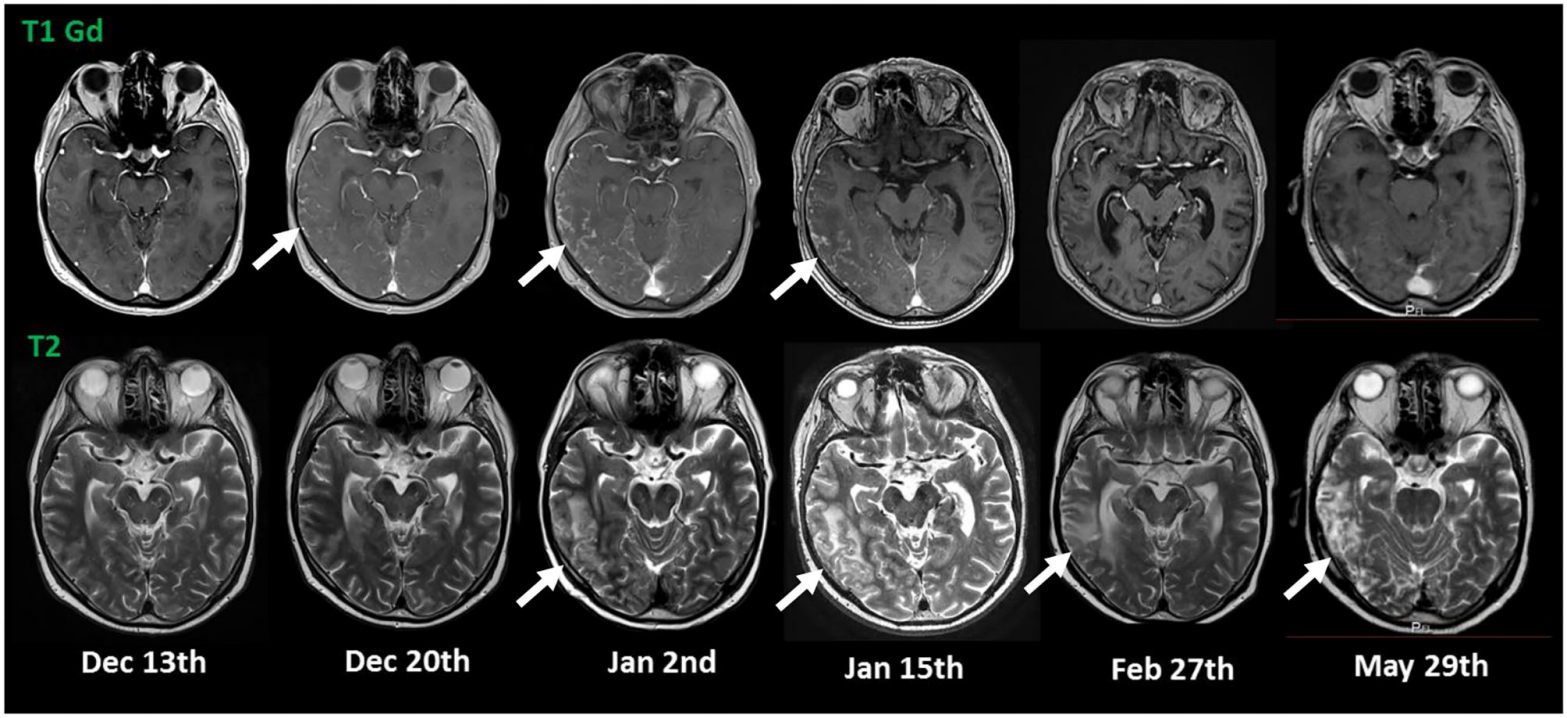
MRI imaging. The initial MRI showed nodular, T1 contrast-enhancing lesions in the right temporo-parietal lobe (indicated by arrows in the upper row) suspicious for sarcoidosis with accompanying edema in T2 (indicated by arrows in the lower row). Discontinuation of dupilumab (Jan 8th) and initiation of a treatment with steroids (Jan 27th) led to quick regression of the nodular lesions and gradual regression of the brain edema.

His cognitive decline was objectified using the Montreal Cognitive Assessment (MoCA), which showed a pathological result of 21/30. His EEG showed a mild diffuse bilateral temporal encephalopathy with a focus on the left side. CSF analysis showed a meningitis syndrome with a lymphocytic pleocytosis (up to 48 cells/μl) and elevated protein (up to 261 mg/dl) with no signs of intrathecal antibody production and normal glucose level. A cranial MRI was performed and showed nodular, primarily sulcal, and pial contrast-enhancing formations. These were predominantly located in the right temporoparietal lobe and accompanied by brain edema, in anatomical correlation to the patient's neuropsychiatric symptoms, as well as his difficulties in orientation. Underlying infectious causes were ruled out through negative PCR results in the CSF for bacterial meningitis, tuberculosis, fungal infections, and viral meningitis including herpesviruses 1 to 6. Serology for cryptococcal and aspergillus antigens was negative. Aerobic and anaerobic bacterial cultures and mycobacterial cultures, as well as fungal cultures in the blood and the CSF, showed no growth. Serology for Lyme's disease, HIV, syphilis, hepatitis B, and C was negative. An extensive search for autoantibodies in the CSF inducing meningoencephalitis syndromes, including antibodies against sodium channels (AMPA1/2, NMDA-receptor, NMDA-NR1-receptor), chloride channels (GABA-B-receptor-1), the potassium channel-complex, including anti-CASPR2 and anti-LGI-1 antibodies, anti-amphiphysin-1, anti-GAD, and anti-Ma1/2-autoantibodies, also showed no positive results. Serological vasculitic parameters were unremarkable. A whole-body PET-CT showed no pathological findings largely ruling out a malignancy or granuloma formation outside the central nervous system. The Angiotensin Converting Enzyme (ACE) levels in blood and CSF were within the normal range. Pausing dupilumab and a 3-day-course of 1 g of methylprednisolone intravenously followed by an oral steroid course for 4 weeks led to quick clinical improvement and regression of his radiological findings on follow-up imaging and normalization of his EEG, as well as his CSF findings. His result on the MoCA also improved within <2 months to 24/30. The atopic dermatitis was treated with topical corticoids. Figure 1 shows the dynamics of his CSF parameters before and after initiation of treatment, whereas Figure 2 shows his findings on the MRI during his first hospitalization and on follow-up.

We postulate the diagnosis of a drug-induced neurosarcoid-like reaction caused by dupilumab, based on the typical imaging and CSF findings, as well as the prompt response to the withdrawal of the responsible drug and corticoid treatment. Normal ACE levels do not rule out the diagnosis given their low sensitivity for sarcoidosis (4).

## Discussion

In February 2020, Belhomme et al. reported the first case of systemic sarcoid-like granulomatosis induced by dupilumab occurring in a 28-year-old patient with atopic dermatitis (5). Drug-induced sarcoid-like reaction (DISRs) is a rare side effect of drugs but is described for immune checkpoint inhibitors, TNF-α antagonists, interferons, and highly active antiretroviral therapy (HAART) (6, 7). Retrospective studies demonstrate its occurrence in 0.2% of people treated with immune checkpoint inhibitors, or 0.04% of people treated with TNF-α inhibitors (6). Treatment of DISR is not always necessary as it can resolve spontaneously or with the withdrawal of the responsible drug. However, if symptoms persist or are severe, corticoids or other drugs used against sarcoidosis are recommended (6). Treatment is necessary depending on the causing agent in 40–60% of the cases and leads to resolution in 60–80% of treated cases (6).

It is not possible to distinguish a DISR from sarcoidosis clinically. Both may present with associations of bilateral hilar adenopathy, cutaneous lesions, uveitis, granulomatous infiltration of scars, hypercalcemia, elevated serum angiotensin-converting enzyme levels, and 18F-fluorodeoxyglucose uptake on PET scans (6). Temporal correlation of symptoms with the initiation of a specific therapy with drugs linked to DISR and resolution of symptoms when the drug is withdrawn are key factors in diagnosing a DISR.

Disturbance of the Th1/Th2 cell equilibrium, as well as their mediators in favor of a Th1 reaction, is thought to be the common underlying pathophysiological mechanism in all these cases (6).

The HAART has been associated with DISR in the setting of a rising CD4 count. An imbalance between Th1 and Th2 cells within this sudden rise with overweighing of the former is discussed to be leading to increased granuloma formation and DISR (8). Likewise, immune checkpoint inhibitors alleviate the immunosuppression of T-cells caused by tumors, prolonging T-cell activation, and restoring T-cell proliferation. Particularly ipilimumab, a CTLA-4 inhibitor linked to DISR, has been shown to lead to T-cell proliferation and an increase in Th1 markers (9, 10). TNF-α inhibitors, although used in the treatment of sarcoidosis, have also been paradoxically shown to cause DISR. In support of this, Etanercept, a soluble TNF-α receptor fusion protein, has been shown to increase the production of IFN-γ, a Th1 mediator, in T-cells (11). Monoclonal TNF-α inhibitors have similarly been shown to increase the Th1/Th2 ratio in the peripheral blood (12). Finally, interferons and particularly IFN-α have been linked to DISR. IFN-α has been associated with Th1 polarization and Th2 inactivation, as well as an increased level of granuloma-promoting cytokines (13). On the other hand, it has been shown that corticoids preferably affect Th1 cells by downregulating the chemokine receptors CCR10 and CXCR3 mainly expressed on Th1 cells, thus, preventing their migration to the site of inflammation. Furthermore, they reduce IFN-γ production in these cells. This may explain the generally good response to corticoids in DISR (14).

Dupilumab inhibits the action of IL-4 and IL-13. IL-4 leads to the differentiation of naive CD4 cells into Th2 cells, whereas IL-13 is a cytokine of the Th2 pathway (15). Dupilumab, blocking the action of these agents, could, therefore, disturb the Th1/Th2 equilibrium leading to an increased Th1-response and granuloma formation, presenting as a DISR.

In summary, it is very important to consider this possible side effect of Dupilumab, especially, given the expected increasing application of the drug in the treatment of atopic dermatitis and asthma.

## Data Availability Statement

The original contributions presented in the study are included in the article/supplementary material, further inquiries can be directed to the corresponding author.

## Ethics Statement

Written informed consent was obtained from the individual(s) for the publication of any potentially identifiable images or data included in this article.

## Author Contributions

LV conceived the original idea. PA, JM, and LV as well as ST and LN with the help of AS treated the patient and performed the diagnostic including imagery. ST and LN wrote the manuscript. LV and AS helped supervise the project. All authors contributed to manuscript revision, read, and approved the submitted version.

## Conflict of Interest

The authors declare that the research was conducted in the absence of any commercial or financial relationships that could be construed as a potential conflict of interest.

## Publisher's Note

All claims expressed in this article are solely those of the authors and do not necessarily represent those of their affiliated organizations, or those of the publisher, the editors and the reviewers. Any product that may be evaluated in this article, or claim that may be made by its manufacturer, is not guaranteed or endorsed by the publisher.
